# Safety and Immunogenicity of a New Inactivated Polio Vaccine Made From Sabin Strains: A Randomized, Double-Blind, Active-Controlled, Phase 2/3 Seamless Study

**DOI:** 10.1093/infdis/jiaa770

**Published:** 2020-12-22

**Authors:** Maria Rosario Capeding, Grace Devota Gomez-Go, Peninnah Oberdorfer, Charissa Borja-Tabora, Lulu Bravo, Josefina Carlos, Auchara Tangsathapornpong, Rattapon Uppala, Kamolwish Laoprasopwattana, Yunjeong Yang, Song Han, Orasri Wittawatmongkol

**Affiliations:** Department of Microbiology, Research Institute for Tropical Medicine, Muntinlupa City, Philippines; Department of Pediatrics, Mary Chiles General Hospital, Manila, Philippines; Division of Pediatric Infectious Diseases, Department of Pediatrics, Faculty of Medicine, Chiang Mai University, Chiang Mai, Thailand; Clinical Research Division, Research Institute for Tropical Medicine, Muntinlupa City, Philippines; Department of Pediatrics, University of the Philippines Manila, Manila, Philippines; Department of Pediatrics, College of Medicine, University of the East-Ramon Magsaysay Memorial Medical Center, Quezon City, Philippines; Department of Pediatrics, Faculty of Medicine, Thammasat University, Pathumthani, Thailand; Department of Pediatrics, Srinagarind Hospital, Khon Kaen University, Khon Kaen, Thailand; Department of Pediatrics, Faculty of Medicine, Prince of Songkla University, Hat Yai, Thailand; Life Sciences, LG Chem, Ltd, Seoul, Republic of Korea; Life Sciences, LG Chem, Ltd, Seoul, Republic of Korea; Department of Pediatrics, Faculty of Medicine Siriraj Hospital, Mahidol University, Bangkok, Thailand

**Keywords:** inactivated poliovirus vaccine, Sabin vaccine, vaccine immunogenicity, safety, health, infant

## Abstract

**Background:**

A new inactivated polio vaccine made from Sabin strains (sIPV) was developed as part of the global polio eradication initiative.

**Methods:**

This randomized, double-blind, active-controlled, phase 2/3 seamless study was conducted in 2 stages. Healthy infants aged 6 weeks were randomly assigned to receive 3 doses of 1 of 4 study vaccines at 6, 10, and 14 weeks of age (336 received low-, middle-, or high-dose sIPV, or conventional IPV [cIPV] in stage I, and 1086 received lot A, B, or C of the selected sIPV dose, or cIPV in stage II). The primary outcome was the seroconversion rate 4 weeks after the third vaccination.

**Results:**

In stage I, low-dose sIPV was selected as the optimal dose. In stage II, consistency among the 3 manufacturing lots of sIPV was demonstrated. The seroconversion rates for Sabin and wild strains of the 3 serotypes after the 3-dose primary series were 95.8% to 99.2% in the lot-combined sIPV group and 94.8% to 100% in the cIPV group, proving the noninferiority of sIPV compared to cIPV. No notable safety risks associated with sIPV were observed.

**Conclusions:**

Low-dose sIPV administered as a 3-dose vaccination was safe and immunogenic compared to cIPV.

**Clinical Trials Registration:**

NCT03169725.

## Teaser

A new sIPV induced high antibody titers and seroconversion for Sabin and wild poliovirus strains; immunogenic noninferiority of sIPV compared to cIPV was demonstrated. No notable safety risks associated with sIPV were observed.

Since the World Health Assembly resolved to eradicate poliomyelitis worldwide in 1988 [1, 2], the number of poliovirus cases worldwide has decreased by more than 99%, from an estimated 350 000 cases in 1988 to 175 reported cases in 2019 [1, 3], but the virus remains endemic in a few countries [1, 4–6]. There is no cure for poliomyelitis; it can only be prevented with poliovirus vaccines, and the worldwide reduction in incidence is largely attributed to vaccines. There are 2 types of vaccines for polio: an inactivated polio vaccine (IPV) and an oral polio vaccine (OPV) [7]. IPV and OPV are both effective in preventing polio disease. However, the use of OPV can cause rare cases of vaccine-associated paralytic poliomyelitis, as well as a significant resurgence of poliomyelitis cases due to circulating vaccine-derived poliovirus [8, 9] and low vaccination coverage in some countries [10, 11]. For this reason, the core of the Polio Eradication and Endgame Strategic Plan implemented by the Global Polio Eradication Initiative is to introduce IPV into routine immunization programs worldwide, as a replacement for OPV [2, 12, 13].

However, the production of conventional IPV (cIPV) with wild poliovirus strains poses higher biosafety risks compared to OPV, primarily because cIPV uses virulent wild poliovirus strains [12, 14, 15]. Therefore, IPV development using attenuated strains such as the Sabin virus, which carries a relatively lower biosafety risk in case of its escape from the manufacturing facility, has been coordinated by the World Health Organization (WHO) [5, 16–18]. In response, Intravacc (Institute for Translational Vaccinology, Bilthoven, Netherlands) developed an IPV from Sabin strains (sIPV) suitable for up-scaling and technology transfer [19]. In sIPV clinical studies conducted by Intravacc [15, 20, 21], sIPV was well tolerated in healthy adults and induced promising immune responses against Sabin and wild strains when administered as 3 primary courses in infants.

LG Chem, Ltd (Seoul, Republic of Korea) received technology transfer from Intravacc to develop a new sIPV (LBVC) in its clinical development program. In this study, we aimed to evaluate the safety and immunogenicity of the new sIPV compared to cIPV given as a 3-dose vaccination in healthy infants, and to determine the optimal dose of sIPV (stage I). Furthermore, in stage II, we aimed to demonstrate lot-to-lot consistency among the 3 manufacturing lots of sIPV and noninferiority of the new sIPV compared to cIPV in terms of immunogenicity, and evaluate the safety of sIPV compared to cIPV.

## METHODS

### Study Design

This study was a multicenter, randomized, double-blind, active-controlled, parallel-group, phase 2/3 seamless study. This study design combined 2 separate trials: a phase 2 study (for dose selection) designated as stage I and a phase 3 study (for immunogenicity confirmation) designated as stage II. At the end of stage I (phase 2), the optimal dose of sIPV was selected based on the results of the interim analysis (stage I). Next, the study proceeded to stage II with the determined optimal dose, new infants were recruited for stage II, and participants in stage I did not contribute to any analysis for stage II. Stage I was conducted at 3 centers in Thailand and the Philippines; stage II was conducted at 10 centers in the same countries. The study was conducted in compliance with all relevant ethical codes and principles of Good Clinical Practice, and it was approved by the independent ethics committee/institutional review board of each study center. The study was registered at ClinicalTrials.gov (NCT03169725).

### Participants

Healthy infants aged 6 weeks (42–56 days) were recruited. Written informed consent was obtained from the parents or legally authorized representatives of the infants before screening the participants. The main exclusion criteria included (1) a medical history of febrile, acute, or progressive illnesses; and (2) known or suspected immune disorders or received immunosuppressive therapy. The full list of inclusion and exclusion criteria is provided in the Supplementary Material.

### Randomization and Blinding

Eligible participants were randomly assigned to 1 of 4 study groups (low-, middle-, or high-dose sIPV group, or a cIPV group in a 1:1:1:1 ratio in stage I [phase 2], and lot A, lot B, or lot C group of the selected sIPV dose in a 1:1:1 ratio or a cIPV group in stage II [phase 3]). Randomization was performed through central randomization using an interactive web response system, and the random sequence was generated by an independent statistician based on a preset block length of 8. Study vaccines were administered by unblinded independent nurses in a separate room. Other site staff, participants, their parents, and the sponsor, including investigators, were all blinded to group assignments.

### Procedures

The test vaccine (LG Chem, Ltd) was a clear, colorless solution (0.5 mL/dose) contained in a transparent vial. It contained inactivated Sabin poliovirus type 1, 2, and 3 strains. Antigen contents of low-, middle-, and high-dose sIPVs studied in stage I were 5, 8, 16; 5, 16, 32; and 5, 32, 32 D-antigen units (DU)/dose (type 1, 2, 3), respectively. The D-antigen contents of sIPV were determined using a validated immunochemical method and calculated using a reference vaccine (cIPV) following WHO recommendations [22]. Low-dose sIPV was selected from stage I, and 3 manufacturing lots (lots A, B, and C) of the selected dose were used in stage II. The control vaccine (Sanofi Pasteur) was a clear, colorless solution (0.5 mL/dose) contained in a transparent prefilled syringe. It contained inactivated Salk poliovirus type 1, 2, and 3 strains. The antigen contents of cIPV were 40, 8, and 32 DU/dose for type 1, 2, and 3.

All randomized participants received 3 doses of the study vaccine at 6, 10, and 14 weeks of age. The interval between doses was at least 28 days to a maximum of 35 days. After the 3-dose primary series, participants visited each study center 28 days after the last vaccination (close-out visit). Follow-up schedules are detailed in Supplementary Figure 1.

Blood samples were obtained by venipuncture from each participant. The microneutralization assay was performed on all blood samples to determine neutralizing antibodies against Sabin and wild serotypes.

Participants were observed for immediate reactions for 30 minutes after each study vaccination. The parents (or legally authorized representatives) of the participants received diary cards, on which they were asked to record solicited adverse events occurring in the participants for 7 days following each study vaccination. Unsolicited adverse events were collected throughout the study period (between each dose and for 4 weeks postdose), and serious adverse events were collected up to 6 months after the last dose of the study vaccine.

Other vaccines scheduled under the National Immunization Program, except for polio vaccines during the study period, were permitted at least 7 days before or after study vaccination.

### Outcomes

The primary outcome in stage I and stage II was the seroconversion rate of neutralizing antibodies against Sabin and wild poliovirus strains of 3 serotypes 4 weeks after the third vaccination. Seroconversion for polio antigen was defined as (1) for participants seronegative at the prevaccination, postvaccination antibody titers of ≥8 (3 log_2_); and (2) for participants seropositive at the prevaccination, a ≥4-fold (2 log_2_) increase in postvaccination antibody titers above the expected maternal antibody titers based on the prevaccination titer declining with a half-life of 28 days. Secondary outcomes were as follows: (1) the seroconversion rate of neutralizing antibodies 4 weeks after the second vaccination, assessed only in stage I; (2) the geometric mean titers (GMTs) of neutralizing antibodies 4 weeks after the second and third vaccinations in stage I and after the third vaccination in stage II; and (3) the seroprotection rate of neutralizing antibodies 4 weeks after the third vaccination, assessed only in stage II. Seroprotection for polio antigen was defined as postvaccination antibody titers of ≥8 (3 log_2_).

Safety endpoints included immediate reactions and solicited adverse events after each study vaccination, as well as unsolicited adverse events. Solicited adverse events were classified as local or systemic reactions; solicited local reactions included pain/tenderness, erythema/redness, and induration/swelling, and solicited systemic reactions included fever, irritability/restlessness, drowsiness/sleepiness, loss of appetite, diarrhea, vomiting, and rash.

### Statistical Analysis

In stage I, no power adjustment based on the number of antigens was planned because this stage was conducted for exploratory purposes. The sample size to assess the seroconversion rate of neutralizing antibodies 4 weeks after the third vaccination was determined using a significance level of 2.5% and a one-sided test. The planned enrollment was 84 infants per group (total 336), which provided 80% power, using an estimated seroconversion rate of 0.95 and a clinically acceptable difference between the sIPV group and cIPV group of 10%, and assuming a dropout rate of 10%.

The sample size in stage II was calculated to demonstrate the lot-to-lot consistency among the 3 manufacturing lots of sIPV and the noninferiority of sIPV compared to cIPV regarding the seroconversion rate of neutralizing antibodies 4 weeks after the third vaccination. The required sample size was 262 infants per sIPV lot group, which provided an overall power of 98% to demonstrate the lot-to-lot consistency among the sIPV lots, using a significance level of 2.5% and 2 one-sided tests, an estimated seroconversion rate of 0.95, and an equivalence margin of 10%. In addition, the required sample size was 180 infants per group, which provided an overall power of 95% to demonstrate the noninferiority of sIPV compared to cIPV, using a significance level of 2.5% and a one-sided test, an estimated seroconversion rate of 0.95, and a noninferiority margin of −10%. Therefore, a sample size of 1076 infants (292 infants in each sIPV lot group and 200 infants in the cIPV group) was required to maintain an overall power of 93% to demonstrate both the lot-to-lot consistency among the sIPV lots and the noninferiority of sIPV compared to cIPV, assuming a dropout rate of 10%.

All participants who received 3 doses of the study vaccine at protocol-defined times and with all antibody titers measured for all serotypes 4 weeks after the third vaccination were included in the immunogenicity analyses (per protocol set). Participants with major protocol deviations that could have affected the immunogenicity of the study vaccines were excluded from the immunogenicity analyses. Safety analyses were performed in all randomized participants who received at least 1 dose of the study vaccine. Immunogenicity and safety analyses were based on the study vaccine administered, regardless of which was assigned at randomization.

The seroconversion rates and seroprotection rates of neutralizing antibodies were calculated, and the between-group differences (sIPV group − cIPV group) and the corresponding 95% confidence intervals (CIs) were summarized. In stage II, the lot-to-lot consistency, in terms of seroconversion rates of neutralizing antibodies 4 weeks after the third vaccination, was tested first between each pair of sIPV lot groups. Equivalence had to be demonstrated in order for data from the 3 sIPV lot groups to be pooled for the noninferiority test. Equivalence would be demonstrated if all 95% CIs for the difference in seroconversion rates were within the equivalence margin (−10% to 10%) simultaneously for each serotype between each pair of sIPV lot groups. Next, the noninferiority of sIPV compared to cIPV was tested. Noninferiority would be demonstrated if the lower limits of all 95% CIs (2-sided) for the difference in seroconversion rates were greater than the noninferiority margin of −10% simultaneously for each serotype between the lot-combined sIPV and cIPV groups. The overall type I error for the immunogenicity hypothesis was controlled at a significance level of .05 (2-sided). The GMTs and mean log_2_ titers of neutralizing antibodies, as well as the reverse cumulative distributions of antibody titers, were also provided. For safety data, descriptive statistics were summarized. Statistical data analyses were performed using SAS version 9.3 or later (SAS Institute).

## RESULTS

In stage I, 336 participants were randomly assigned to 1 of 4 study groups between 31 May and 15 August 2017 (Supplementary Figure 2). Demographics and baseline characteristics of the participants were well balanced among the groups (Supplementary Table 1).

After the 3-dose primary series, seroconversion rates for each serotype of the sIPV-dose groups were 90.4% to 100%, similar to those in the cIPV group (97.6% to 100%; Supplementary Table 2). The seroconversion rate for wild strains of serotype 1 in the low- and middle-dose sIPV groups was significantly lower than that in the cIPV group but achieved more than 90% seroconversion in the low- and middle-dose sIPV groups.

The incidence of solicited adverse events in the high-dose group was significantly higher than that in the cIPV group but the incidence in the low- and middle-dose groups was similar, and there was no significant difference with the cIPV group (Supplementary Table 3). Most of the solicited adverse events were mild in severity.

Based on results for immunogenicity and safety, low-dose sIPV was selected as the optimal dose.

**Figure 1.**
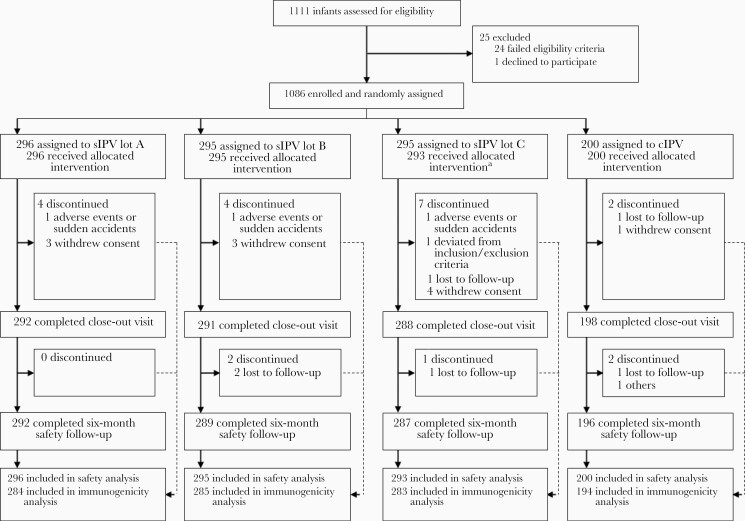
Subject disposition in stage II. ^a^Of the randomized participants, 2 in the sIPV lot C group dropped out of the study before receiving the first dose of the study vaccine. Abbreviations: cIPV, conventional inactivated polio vaccine; sIPV, inactivated polio vaccine made from Sabin strains.

In stage II, 1086 participants were randomly assigned to 1 of 4 study groups between 3 September and 15 October 2018 (Figure 1). The ratio of male to female infants was similar, and baseline characteristics of participants were well balanced among the groups (Table 1).

**Table 1.** Baseline Characteristics in Stage II

Characteristics	sIPV						
	sIPV Lot A	sIPV Lot B	sIPV Lot C	Combined sIPV	cIPV	*P* Value	Difference or Ratio of GMT^a^ (95% CI) Combined sIPV vs cIPV
Safety set							
Participants, No.	296	295	293	884	200	…	…
Age, d, mean (SD)	47.4 (4.33)	46.9 (4.20)	47.6 (4.39)	47.3 (4.31)	47.3 (4.40)	.2805^b^	…
Sex						.3288^c^	
Male, No. (%)	149 (50.3)	144 (48.8)	164 (56.0)	457 (51.7)	101 (50.5)	…	…
Female, No. (%)	147 (49.7)	151 (51.2)	129 (44.0)	427 (48.3)	99 (49.5)	…	…
Height, cm, mean (SD)	55.09 (2.078)	54.99 (2.311)	55.26 (2.223)	55.08 (2.262)	55.11 (2.266)	.3329^b^	…
Weight, kg, mean (SD)	4.67 (0.524)	4.71 (0.533)	4.73 (0.586)	4.72 (0.563)	4.72 (0.561)	.7179^b^	…
Per protocol set							
Participants, No.	284	285	283	852	194	…	…
Age, d, mean (SD)	47.4 (4.32)	46.8 (4.17)	47.7 (4.40)	47.3 (4.31)	47.4 (4.40)	.1641^b^	…
Sex						.2621^c^	
Male, No. (%)	139 (48.9)	140 (49.1)	159 (56.2)	438 (51.4)	97 (50.0)	…	…
Female, No. (%)	145 (51.1)	145 (50.9)	124 (43.8)	414 (48.6)	97 (50.0)	…	…
Height, cm, mean (SD)	54.97 (2.302)	55.28 (2.203)	55.11 (2.254)	55.12 (2.254)	55.12 (2.085)	.2712^b^	…
Weight, kg, mean (SD)	4.70 (0.537)	4.73 (0.585)	4.73 (0.561)	4.72 (0.561)	4.68 (0.525)	.8060^b^	…
Sabin type 1							
Seropositive rate, No. (%)	119 (41.9)	133 (46.7)	124 (43.8)	376 (44.1)	90 (46.4)	…	−2.3 (−10.0 to 5.4)
GMT	12.31	13.11	12.97	12.79	12.67	…	1.01^a^ (.83 to 1.23)
Mean log_2_ titers (SD)	3.62 (1.846)	3.71 (1.836)	3.70 (1.813)	3.68 (1.830)	3.66 (1.673)	…	0.01 (−.27 to .30)
Sabin type 2							
Seropositive rate, No. (%)	151 (53.2)	160 (56.1)	149 (52.7)	460 (54.0)	102 (52.6)	…	1.4 (−6.3 to 9.2)
GMT	13.52	14.45	13.55	13.84	11.64	…	1.19^a^ (.99 to 1.43)
Mean log_2_ titers (SD)	3.76 (1.715)	3.85 (1.804)	3.76 (1.716)	3.79 (1.744)	3.54 (1.392)	…	0.25 (−.01 to .51)
Sabin type 3							
Seropositive rate, No. (%)	76 (26.8)	81 (28.4)	69 (24.4)	226 (26.5)	47 (24.2)	…	2.3 (−4.8 to 8.6)
GMT	9.88	10.80	9.74	10.13	8.48	…	1.19^a^ (.99 to 1.45)
Mean log_2_ titers (SD)	3.30 (1.772)	3.43 (1.982)	3.28 (1.788)	3.34 (1.849)	3.08 (1.379)	…	0.26 (−.02 to .53)
Wild type 1 (Mahoney)							
Seropositive rate, No. (%)	92 (32.4)	88 (30.9)	92 (32.5)	272 (31.9)	65 (33.5)	…	−1.6 (−9.1 to 5.5)
GMT	8.60	8.29	9.07	8.65	8.43	…	1.03^a^ (.92 to 1.15)
Mean log_2_ titers (SD)	3.10 (1.062)	3.05 (0.946)	3.18 (1.150)	3.11 (1.056)	3.08 (0.971)	…	0.04 (−.13 to .20)
Wild type 2 (MEF-1)							
Seropositive rate, No. (%)	168 (59.2)	172 (60.4)	163 (57.6)	503 (59.0)	117 (60.3)	…	−1.3 (−8.7 to 6.5)
GMT	12.70	13.15	13.24	13.03	12.05	…	1.08^a^ (.95 to 1.24)
Mean log_2_ titers (SD)	3.67 (1.242)	3.72 (1.254)	3.73 (1.306)	3.70 (1.267)	3.59 (1.115)	…	0.11 (−.08 to .31)
Wild type 3 (Saukett)							
Seropositive rate, No. (%)	56 (19.7)	56 (19.6)	44 (15.5)	156 (18.3)	35 (18.0)	…	0.3 (−6.2 to 5.8)
GMT	7.35	7.10	6.98	7.14	6.83	…	1.05^a^ (.96 to 1.14)
Mean log_2_ titers (SD)	2.88 (0.889)	2.83 (0.742)	2.80 (0.848)	2.84 (0.828)	2.77 (0.661)	…	0.06 (−.06 to .19)

Abbreviations: CI, confidence interval; cIPV, conventional inactivated polio vaccine; GMT, geometric mean titer; SD, standard deviation; sIPV, inactivated polio vaccine made from Sabin strains.

^a^The ratio of GMT was calculated as the GMT in the combined sIPV group divided by the GMT in the cIPV group.

^b^ *P* value among the 4 study groups was obtained from Kruskal-Wallis test.

^c^ *P* value among the 4 study groups was obtained from χ ^2^ test.

After the 3-dose primary series, the seroconversion rates for Sabin and wild strains of each of the 3 serotypes in each sIPV lot group were 95.4% to 99.6%, and the 95% CIs for the difference in seroconversion rates for each serotype between each pair of the 3 lot groups were within the equivalence margin (−10% to 10%), demonstrating the lot-to-lot equivalence among the sIPV lots (Table 2). In addition, seroconversion rates for each serotype of the lot-combined sIPV group were 95.8% to 99.2%, and the lower limits of the 95% CIs for the difference between the lot-combined sIPV and cIPV groups were greater than the noninferiority margin (−10%), confirming the noninferiority of sIPV compared to cIPV (Table 2).

**Table 2.** Seroconversion Rates After the Third Vaccination in Stage II (Per Protocol Set)

Type	sIPV			Lot-to-Lot Consistency Among the 3 Lots of sIPV					
	sIPV Lot A (n = 284)	sIPV Lot B (n = 285)	sIPV Lot C (n = 283)	Difference (95% CI) Lot A − Lot B	Difference (95% CI) Lot A − Lot C	Difference (95% CI) Lot B − Lot C	Combined sIPV (n = 852)	cIPV (n = 194)	Difference (95% CI) sIPV − cIPV
Sabin type 1	275 (96.8)	279 (97.9)	276 (97.5)	−1.1 (−4.0 to 1.8)	−0.7 (−3.7 to 2.3)	0.4 (−2.4 to 3.2)	830 (97.4)	187 (96.4)	1.0 (−1.2 to 4.8)
Sabin type 2	278 (97.9)	274 (96.1)	278 (98.2)	1.7 (−1.2 to 4.9)	−0.3 (−3.0 to 2.2)	−2.1 (−5.2 to .8)	830 (97.4)	184 (94.8)	2.6 (−.1 to 6.7)
Sabin type 3	273 (96.1)	272 (95.4)	271 (95.8)	0.7 (−2.8 to 4.2)	0.4 (−3.1 to 3.8)	−0.3 (−3.9 to 3.2)	816 (95.8)	190 (97.9)	−2.2 (−4.2 to 1.2)
Wild type 1 (Mahoney)	277 (97.5)	276 (96.8)	272 (96.1)	0.7 (−2.2 to 3.7)	1.4 (−1.6 to 4.6)	0.7 (−2.5 to 4.0)	825 (96.8)	194 (100)	−3.2 (−4.6 to −1.0)
Wild type 2 (MEF-1)	280 (98.6)	280 (98.2)	278 (98.2)	0.3 (−2.0 to 2.8)	0.4 (−2.0 to 2.8)	0.0 (−2.5 to 2.5)	838 (98.4)	193 (99.5)	−1.1 (−2.3 to 1.3)
Wild type 3 (Saukett)	281 (98.9)	284 (99.6)	280 (98.9)	−0.7 (−2.7 to 1.0)	0.0 (−2.1 to 2.1)	0.7 (−1.0 to 2.7)	845 (99.2)	193 (99.5)	−0.3 (−1.3 to 2.1)

Data are No. (%) unless otherwise indicated.

Abbreviations: CI, confidence interval; cIPV, conventional inactivated polio vaccine; sIPV, inactivated polio vaccine made from Sabin strains.

**Figure 2.**
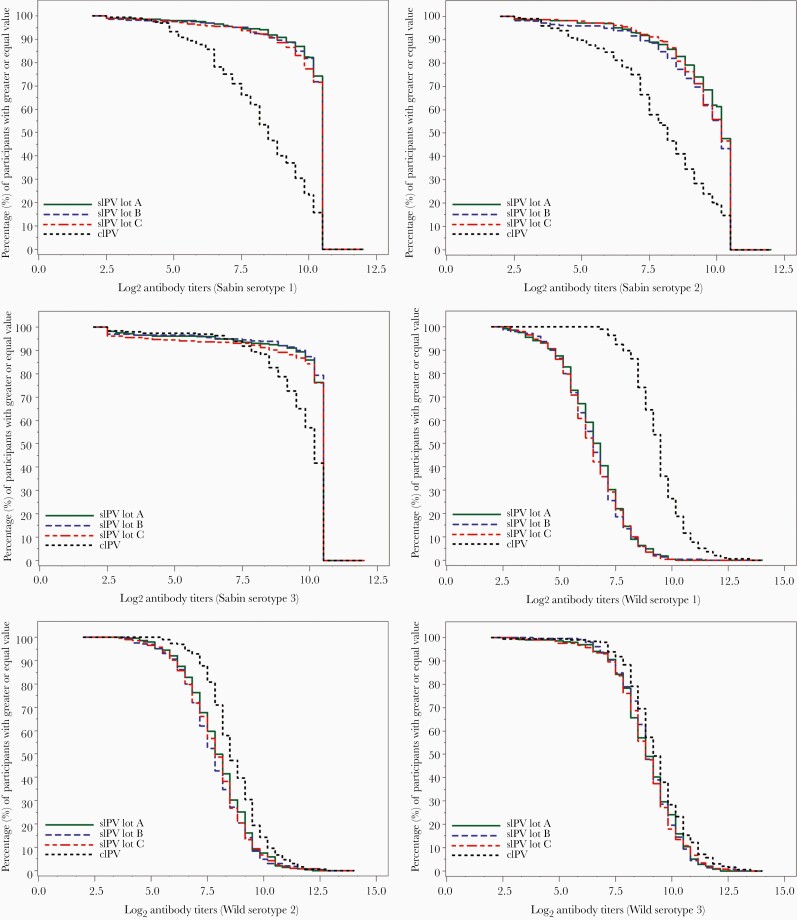
Reverse cumulative distributions of antibody titers after the third vaccination in stage II. Abbreviations: cIPV, conventional inactivated polio vaccine; sIPV, inactivated polio vaccine made from Sabin strains.

The GMTs for each serotype increased significantly after the third vaccination in all treatment groups compared to prevaccination (Table 3), and the corresponding reverse cumulative distribution curves of antibody titers are illustrated in Figure 2.

**Table 3.** Seroprotection Rates and GMTs After the Third Vaccination in Stage II (Per Protocol Set)

Type	sIPV					
	sIPV Lot A (n = 284)	sIPV Lot B (n = 285)	sIPV Lot C (n = 283)	Combined sIPV (n = 852)	cIPV (n = 194)	Treatment Difference or Ratio of GMT^a^ (95% CI) Combined sIPV vs cIPV
Sabin type 1						
Seroprotection rate, No. (%)	281 (98.9)	281 (98.6)	280 (98.9)	842 (98.8)	193 (99.5)	−0.7 (−1.7 to 1.7)
GMT	1051.97	1013.79	985.88	1016.89	307.74	3.30^a^ (2.82 to 3.87)
GMT ratio^b^ (95% CI)	85.48 (69.49 to 105.15)	77.34 (62.90 to 95.10)	75.99 (61.91 to 93.26)	79.50 (70.60 to 89.51)	24.29 (18.78 to 31.42)	…
Mean log_2_ titers (SD)	10.04 (1.272)	9.99 (1.384)	9.95 (1.383)	9.99 (1.346)	8.27 (1.840)	1.72 (1.50 to 1.95)
Sabin type 2						
Seroprotection rate, No. (%)	280 (98.6)	280 (98.2)	279 (98.6)	839 (98.5)	192 (99.0)	−0.5 (−1.8 to 2.2)
GMT	778.00	669.91	754.36	732.48	246.49	2.97^a^ (2.49 to 3.55)
GMT ratio^b^ (95% CI)	57.55 (46.91 to 70.59)	46.35 (36.89 to 58.24)	55.67 (46.03 to 67.32)	52.94 (46.96 to 59.69)	21.17 (16.25 to 27.58)	…
Mean log_2_ titers (SD)	9.60 (1.476)	9.39 (1.717)	9.56 (1.450)	9.52 (1.553)	7.95 (1.973)	1.57 (1.32 to 1.83)
Sabin type 3						
Seroprotection rate, No. (%)	277 (97.5)	276 (96.8)	272 (96.1)	825 (96.8)	191 (98.5)	−1.6 (−3.4 to 1.4)
GMT	1025.00	1040.42	925.55	995.80	768.62	1.30^a^ (1.09 to 1.54)
GMT ratio^b^ (95% CI)	103.75 (82.97 to 129.72)	96.32 (76.77 to 120.84)	95.04 (74.76 to 120.83)	98.30 (86.11 to 112.21)	90.64 (73.41 to 111.91)	…
Mean log_2_ titers (SD)	10.00 (1.566)	10.02 (1.568)	9.85 (1.842)	9.96 (1.663)	9.59 (1.454)	0.37 (.12 to .63)
Wild type 1 (Mahoney)						
Seroprotection rate, No. (%)	280 (98.6)	279 (97.9)	279 (98.6)	838 (98.4)	194 (100)	−1.6 (−2.7 to .4)
GMT	95.04	87.90	86.95	89.90	665.34	0.14^a^ (.12 to .16)
GMT ratio^b^ (95% CI)	11.05 (9.43 to 12.95)	10.60 (9.10 to 12.35)	9.59 (8.18 to 11.25)	10.40 (9.50 to 11.38)	78.92 (67.62 to 92.11)	…
Mean log_2_ titers (SD)	6.57 (1.455)	6.46 (1.439)	6.44 (1.419)	6.49 (1.437)	9.38 (1.125)	−2.89 (−3.10 to −2.67)
Wild type 2 (MEF-1)						
Seroprotection rate, No. (%)	284 (100)	285 (100)	283 (100)	852 (100)	194 (100)	0 (…)
GMT	252.73	220.45	232.40	234.80	406.91	0.58^a^ (.49 to .67)
GMT ratio^b^ (95% CI)	19.90 (16.80 to 23.58)	16.76 (14.08 to 19.96)	17.55 (14.59 to 21.11)	18.02 (16.28 to 19.95)	33.77 (27.76 to 41.08)	…
Mean log_2_ titers (SD)	7.98 (1.415)	7.78 (1.472)	7.86 (1.469)	7.88 (1.453)	8.67 (1.272)	−0.79 (−1.02 to −.57)
Wild type 3 (Saukett)						
Seroprotection rate, No. (%)	284 (100)	285 (100)	282 (99.6)	851 (99.9)	193 (99.5)	0.4 (−.3 to 2.7)
GMT	492.50	503.83	467.42	487.72	643.54	0.76^a^ (.65 to .88)
GMT ratio^b^ (95% CI)	67.00 (57.45 to 78.14)	70.98 (62.11 to 81.13)	66.94 (57.54 to 77.87)	68.29 (62.77 to 74.29)	94.25 (80.18 to 110.77)	…
Mean log_2_ titers (SD)	8.94 (1.422)	8.98 (1.264)	8.87 (1.472)	8.93 (1.388)	9.33 (1.338)	−0.40 (−.62 to −.18)

Abbreviations: sIPV, inactivated polio vaccine made from Sabin strains; cIPV, conventional inactivated polio vaccine; GMT, geometric mean titer; CI, confidence interval; SD, standard deviation.

^a^The ratio of GMT was calculated as the GMT in the combined sIPV group divided by the GMT in the cIPV group.

^b^GMT ratio was calculated as the GMT postvaccination divided by the GMT prevaccination.

The incidence of solicited adverse events in the lot-combined sIPV group was 68.1%, which was slightly higher than the 60.0% in the cIPV group (Table 4). Most solicited adverse events were mild in severity, and participants fully recovered within 7 days of vaccination without additional treatment. The incidence of unsolicited adverse events was similar between the groups. The most frequently reported unsolicited adverse event was upper respiratory tract infection, which was not related to the study vaccine. The most frequent immediate reaction occurred in 6 cases of erythema/redness. The most reported serious adverse event was pneumonia, followed by gastroenteritis. There were no vaccine-related serious adverse events in any of the groups. No clinically significant results were found on physical examination.

**Table 4.** Adverse Events in Stage II (Safety Set)

Adverse Events	Combined sIPV (n = 884), No. (%)	cIPV (n = 200), No. (%)	Difference (95% CI)
Any adverse events up to close-out visit^a^	772 (87.3)	165 (82.5)	4.8 (−.4 to 11.0)
Immediate reactions	4 (0.5)	3 (1.5)	−1.0 (−3.9 to .2)
Solicited immediate reactions	4 (0.5)	3 (1.5)	…
Solicited local immediate reactions	3 (0.3)	3 (1.5)	…
Solicited systemic immediate reactions	1 (0.1)	1 (0.5)	…
Unsolicited immediate reactions	0	0	…
Solicited adverse events	602 (68.1)	120 (60.0)	8.1 (.8 to 15.6)
Solicited adverse drug reactions	580 (65.6)	116 (58.0)	7.6 (.3 to 15.2)
Solicited local adverse events	350 (39.6)	60 (30.0)	…
Pain/tenderness	332 (37.6)	54 (27.0)	…
Erythema/redness	48 (5.4)	13 (6.5)	…
Induration/swelling	38 (4.3)	7 (3.5)	…
Solicited systemic adverse events	535 (60.5)	108 (54.0)	…
Fever	51 (5.8)	10 (5.0)	…
Irritability/restlessness	403 (45.6)	81 (40.5)	…
Drowsiness/sleepiness	258 (29.2)	51 (25.5)	…
Loss of appetite	123 (13.9)	29 (14.5)	…
Diarrhea	126 (14.3)	29 (14.5)	…
Vomiting	135 (15.3)	27 (13.5)	…
Rash	61 (6.9)	18 (9.0)	…
Unsolicited adverse events	544 (61.5)	121 (60.5)	1.0 (−6.2 to 8.6)
Unsolicited adverse drug reactions	1 (0.1)	1 (0.5)	−0.4 (−2.7 to .3)
Any serious adverse events up to close-out visit^a^	31 (3.5)	6 (3.0)	0.5 (−3.0 to 2.7)
Solicited serious adverse events	0	0	…
Unsolicited serious adverse events	31 (3.5)	6 (3.0)	…
Unsolicited serious adverse drug reactions	0	0	…
Any serious adverse events after close-out visit^a^	29 (3.3)	8 (4.0)	−0.7 (−4.5 to 1.7)

Abbreviations: CI, confidence interval; cIPV, conventional inactivated polio vaccine; sIPV, inactivated polio vaccine made from Sabin strains.

^a^The close-out visit was 1 month after the last vaccination.

## DISCUSSION

This phase 2/3 seamless study was conducted in 2 independent stages. In stage I, 336 healthy infants received 3 doses of sIPV (low-, middle-, or high-dose sIPV), or cIPV. The seroconversion rates for Sabin and wild strains of the 3 serotypes of the sIPV dose groups were similar to those in the cIPV group. No safety concerns were detected in the sIPV dose groups, and low-dose sIPV (5, 8, 16 DU/dose for type 1, 2, 3) was selected as the optimal dose. In stage II, 1086 healthy infants received either 1 of 3 lots of sIPV or cIPV at 6, 10, and 14 weeks of age. The seroconversion rates for each serotype of each sIPV lot group ranged from 95% to 100%, confirming the lot-to-lot consistency of sIPV. The seroconversion rates for each serotype of the lot-combined sIPV group were 96% to 99%, comparable with those of the cIPV group, demonstrating the noninferiority of sIPV compared to cIPV.

In this study, cross-neutralization assays were performed for the entire study population. After the 3-dose primary series, the GMTs for Sabin strains were significantly higher in the lot-combined sIPV group than in the cIPV group, and GMTs for wild strains were significantly lower in the lot-combined sIPV group than in the cIPV group. This tendency was seen more clearly in the reverse cumulative distributions of antibody titers. This can be considered in the same context as the previous finding that neutralizing antibody titer for homologous strains is higher than that against heterologous strains [6]. Nonetheless, GMTs for both Sabin and wild strains of serotypes significantly increased after 3 vaccinations compared to prevaccination in all the treatment groups, and sIPV induced more than 90% seroconversion. Furthermore, the seroconversion rates in this study were similar to those in other recent sIPV studies, in which seroconversion for Sabin strains was achieved in 95% to 100% of infants [7, 14], and to those in cIPV studies, in which seroconversion for wild strains was achieved in 86% to 100% of infants [23–25]. Therefore, the quantitative difference between the neutralizing antibody titers for Sabin and wild strains in our study has minimal clinical significance [5], suggesting that sIPV induced good immune responses for both the Sabin and wild poliovirus strains.

The safety profile of sIPV was comparable to that of cIPV. Although the incidence of solicited adverse events in the lot-combined sIPV group was 68.1%, slightly higher than the 60.0% in the cIPV group, no clinically significant differences were found between the groups when considering the severity, outcome, and duration of solicited adverse events. The incidence of solicited adverse events in our study was similar to those in other IPV studies, in which solicited adverse events were reported in up to 47.5% to 96.6% of infants [7, 14, 26]. In addition, no vaccine-related serious adverse events were reported in any of the treatment groups in this study, and no other safety issues were identified.

There are other licensed sIPVs in Japan and China [5], but with the plan for OPV withdrawal, there is now more demand than ever to produce new IPVs to relieve the global shortage of IPV. In addition, the development of more affordable IPVs, including sIPV for low- and middle-income countries, is required by the WHO [5, 16]. In our study, sIPV induced good immune responses against both Sabin and wild poliovirus strains compared to cIPV, a very promising finding in view of the limited prior evidence for using sIPV as an alternative to cIPV. Furthermore, the use of sIPV to replace OPV will eliminate the risk of creating new circulating vaccine-derived poliovirus following vaccination. In addition to the multiple candidates for novel polio vaccines, we expect that the use of this new sIPV will contribute to relieving the current global shortage of IPV.

This study had some limitations. First, simultaneous administration with other vaccines under the National Immunization Program, to obtain more accurate safety and immunogenicity information for sIPV, was not allowed. Instead, staggered administration was allowed at least 7 days before or after the study vaccination. However, clinically relevant interference has not been reported when IPV is used with other vaccines [10]. Therefore, we expect no interference with coadministration of sIPV and other vaccines required for a primary immunization series. Second, this study did not include a booster dose in the vaccination schedule; therefore, we could not evaluate the persistence of neutralizing antibodies after completion of the 3-dose primary series. However, the immunogenicity of a booster dose of sIPV was evaluated by Resik et al in a follow-up study to a phase 1 trial in adults who had received multiple doses of OPV during childhood, and it was suggested that historical data on long-term persistence and decay of cIPV could be widely acceptable for sIPV [27]. Further studies may be required to evaluate long-term persistence for a booster dose as needed.

In conclusion, this study demonstrated that sIPV administered as a 3-dose vaccination in healthy infants is comparable to cIPV in terms of immunogenicity, and no notable safety risks associated with sIPV were observed. Therefore, sIPV is expected to play a critical role in polio eradication.

## Supplementary Data

Supplementary materials are available at *The Journal of Infectious Diseases* online. Consisting of data provided by the authors to benefit the reader, the posted materials are not copyedited and are the sole responsibility of the authors, so questions or comments should be addressed to the corresponding author.

jiaa770_suppl_Supplementary_Figure_S1

jiaa770_suppl_Supplementary_Figure_S2

jiaa770_suppl_Supplementary_Material

jiaa770_suppl_Supplementary_Table_S1

jiaa770_suppl_Supplementary_Table_S2

jiaa770_suppl_Supplementary_Table_S3

## Notes

***Acknowledgments.*** We acknowledge the support of the study participants and their parents. We also thank all investigators and site staff who devoted their time and energy to this study, the staff of the Centers for Disease Control and Prevention for analyzing serological samples using the microneutralization assay, and Yunae Eom of LG Chem, Ltd for writing assistance in preparing the manuscript. We are grateful to the data monitoring committee, comprising Salvacion Rodriguez Gatchalian, Ian Zhang, and Michael Desmond Nissen, for monitoring safety and immunogenicity data and for making recommendations for dose selection.

***Author contributions***. M. R. C., G. D. G.-G., P. O., C. B.-T., L. B., J. C., A. T., R. U., K. L., and O. W. conducted the study and contributed to data acquisition. M. R. C. interpreted the data and wrote the manuscript. Y. Y. contributed to the study design and developed the study protocol. S. H. contributed to data analysis. All authors have read and approved the final manuscript.

***Disclaimer***. The findings and conclusions in this report are those of the authors and do not necessarily represent the official position of the Bill and Melinda Gates Foundation or any other collaborating institution. The study funder (Bill and Melinda Gates Foundation) had no role in the study design, data collection, data analysis, or data interpretation, and reviewed the final manuscript of the report. The sponsor (LG Chem, Ltd) participated in the study design, data analysis, data interpretation, and writing of the report but had no role in the data collection.

***Financial support.*** This work was supported by the Bill and Melinda Gates Foundation and LG Chem, Ltd.

***Potential conflicts of interest.*** M. R. C., G. D. G.-G., P. O., C. B.-T., L. B., J. C., A. T., R. U., K. L., and O. W. have received research grants from LG Chem, Ltd. Y. Y. and S. H. are employees of LG Chem, Ltd. All authors have submitted the ICMJE Form for Disclosure of Potential Conflicts of Interest. Conflicts that the editors consider relevant to the content of the manuscript have been disclosed.
