# Supplementary material for: Safety and Immunogenicity of a New Inactivated Polio Vaccine Made From Sabin Strains: A Randomized, Double-Blind, Active-Controlled, Phase 2/3 Seamless Study
Source: J Infect Dis. 2020 Dec 22;226(2):308–18. doi: 10.1093/infdis/jiaa770 (PMC9400411; doi:10.1093/infdis/jiaa770)
Supplement: jiaa770_suppl_Supplementary_Figure_S1 [file jiaa770_suppl_supplementary_figure_s1.docx]

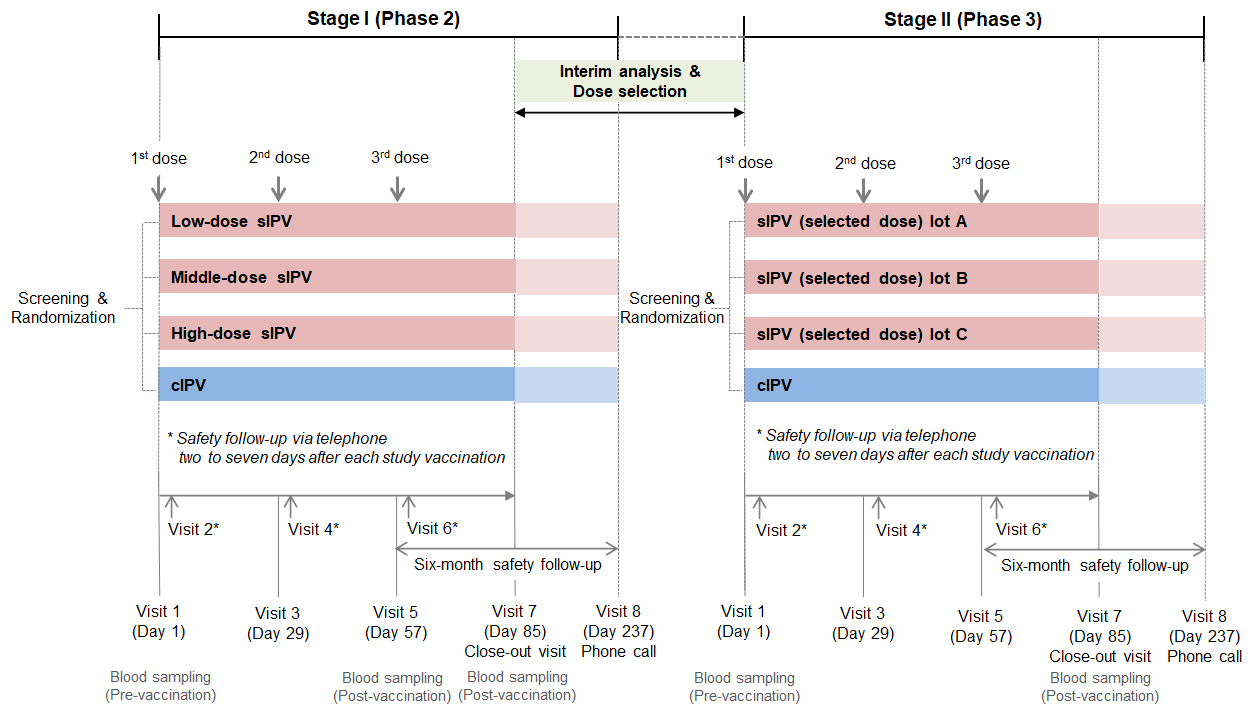


## **Figure S1. Study Design Diagram**

All randomized participants received three doses of the study vaccine at 6, 10, and 14 weeks of age. The study vaccines were administered intramuscularly in the anterolateral aspect of the thigh, and administration schedule was based on the actual age of the participant at the time of the first vaccination. The interval between doses was at least 28 days to a maximum of 35 days.

Blood samples (approximately 3 mL) were obtained by venipuncture from each participant: thrice in Stage I and twice in Stage II, namely, at the pre-vaccination (in Stage I and Stage II), 4 weeks after the second vaccination (only in Stage I for exploratory purposes), and 4 weeks after the third vaccination (in Stage I and Stage II). The microneutralization assay was performed on all blood samples to determine the neutralizing antibodies against Sabin and wild serotypes at the Centers for Disease Control and Prevention (Atlanta, GA, USA) and Viroclinics Biosciences B.V. (Rotterdam, AK, Netherlands; analyzed against only wild serotypes in Stage II).

Safety data were periodically reviewed by the independent data monitoring committee (IDMC), and immunogenicity data were also reviewed by the IDMC to obtain recommendations for optimal dose selection in Stage I.

Immunogenicity and safety data collected during follow-up up to 1 month after the last vaccination (Visit 7: Close-out) in Stage I were analyzed at the interim analysis; 6-month safety follow-up for Stage I could progress concurrently with Stage II, depending on the interim analysis schedule.
