# Supplementary material for: Safety and Immunogenicity of a New Inactivated Polio Vaccine Made From Sabin Strains: A Randomized, Double-Blind, Active-Controlled, Phase 2/3 Seamless Study
Source: J Infect Dis. 2020 Dec 22;226(2):308–18. doi: 10.1093/infdis/jiaa770 (PMC9400411; doi:10.1093/infdis/jiaa770)
Supplement: jiaa770_suppl_Supplementary_Figure_S2 [file jiaa770_suppl_supplementary_figure_s2.docx]

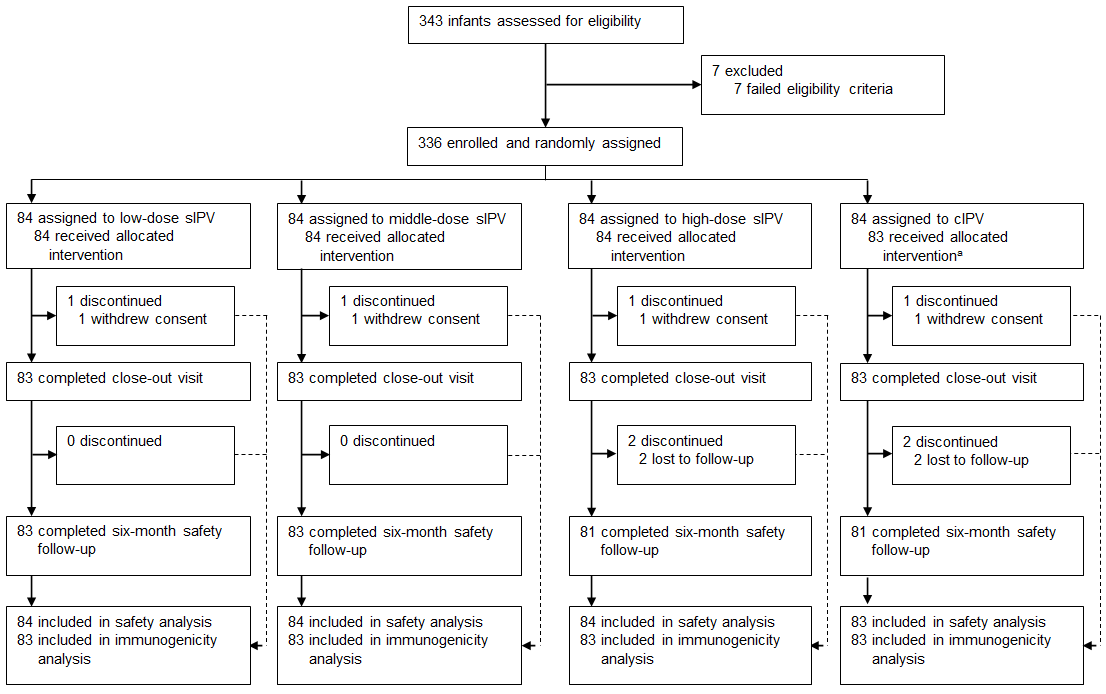


## **Figure S2. Subject Disposition in Stage I**

^a^ Of the randomized participants, one in the cIPV group dropped out of the study before receiving the first dose of the study vaccine.

Abbreviations: sIPV, inactivated polio vaccine made from Sabin strains; cIPV, conventional inactivated polio vaccine.
