# Supplementary material for: Safety and Immunogenicity of a New Inactivated Polio Vaccine Made From Sabin Strains: A Randomized, Double-Blind, Active-Controlled, Phase 2/3 Seamless Study
Source: J Infect Dis. 2020 Dec 22;226(2):308–18. doi: 10.1093/infdis/jiaa770 (PMC9400411; doi:10.1093/infdis/jiaa770)
Supplement: jiaa770_suppl_Supplementary_Material [file jiaa770_suppl_supplementary_material.docx]

**Supplementary Material**

**Study Population**

**Inclusion Criteria**

To participate in the study, all participants had to fulfil the inclusion criteria:

1. Healthy male or female infants who were at least 42 days (6 weeks) of age and not more than 56 days (8 weeks) of age at the time of enrollment.
2. Born at full term of pregnancy (gestational age ≥ 37 weeks).
3. Bodyweight ≥ 3.2 kg at the time of screening.
4. Born to human immunodeficiency virus negative mother.
5. The parents or legally authorized representative (LAR) were able to understand and comply with the planned study procedures.
6. Signed informed consent by a participant’s parents or LAR.

**Exclusion Criteria**

To participate in the study, none of the following criteria could be applied to any participant:

1. Previously received any polio vaccines (oral polio vaccine or inactivated polio vaccine).
2. History of previous or concurrent vaccinations other than hepatitis B, *Bacillus Calmette-Guerin*, *Haemophilus influenzae* type b, diphtheria, tetanus, pertussis, rotavirus vaccine, and pneumococcal conjugate vaccine.
3. History of bleeding disorder contraindicating intramuscular injection.
4. Experienced fever ≥ 38°C/100.4°F within the past 3 days before screening.
5. Received immunoglobulin or blood-derived product since birth.
6. History of allergy reactions to any vaccine components, including excipients and preservatives (e.g neomycin, streptomycin, and polymyxin B).
7. Had a known or suspected immune disorder, or had received immunosuppressive therapy.
8. History of poliomyelitis.
9. History of any neurological disorders or seizures.
10. Known or suspected febrile, acute, or progressive illness.
11. Household contact with and/or intimate exposure to, in the previous 30 days, an individual with poliomyelitis.
12. Participated in another interventional trial within 30 days before enrollment or simultaneous participation in another clinical study.
13. Infants whose families were planning to leave the area of the study site before the end of the study period.
14. Infants considered unsuitable for the clinical study by the investigator.
