# Supplementary material for: Safety and Immunogenicity of a New Inactivated Polio Vaccine Made From Sabin Strains: A Randomized, Double-Blind, Active-Controlled, Phase 2/3 Seamless Study
Source: J Infect Dis. 2020 Dec 22;226(2):308–18. doi: 10.1093/infdis/jiaa770 (PMC9400411; doi:10.1093/infdis/jiaa770)
Supplement: jiaa770_suppl_Supplementary_Table_S1 [file jiaa770_suppl_supplementary_table_s1.docx]

## **Table S1. Baseline Characteristics in Stage I**

|  | **sIPV** | | | **cIPV** | ***P*-value** | **Difference or ratio of GMT^a^ (95% CI)** | | |
| --- | --- | --- | --- | --- | --- | --- | --- | --- |
|  | **Low-dose sIPV** | **Middle-dose sIPV** | **High-dose sIPV** |  |  | **Low-dose sIPV vs cIPV** | **Middle-dose sIPV vs cIPV** | **High-dose sIPV vs cIPV** |
| Safety set |  |  |  |  |  |  |  |  |
| Participants, no. | 84 | 84 | 84 | 83 | ·· | ·· | ·· | ·· |
| Age (days), mean (SD) | 48.4 (4.22) | 48.0 (4.41) | 49.0 (4.15) | 48.1 (4.35) | 0.3861^b^ | ·· | ·· | ·· |
| Sex |  |  |  |  | 0.4942^c^ |  |  |  |
| Male, no. (%) | 45 (53.6%) | 37 (44.0%) | 40 (47.6%) | 45 (54.2%) | ·· | ·· | ·· | ·· |
| Female, no. (%) | 39 (46.4%) | 47 (56.0%) | 44 (52.4%) | 38 (45.8%) | ·· | ·· | ·· | ·· |
| Height (cm), mean (SD) | 55.28 (2.204) | 55.45 (2.161) | 55.65 (1.944) | 55.71 (2.447) | 0.4320^b^ | ·· | ·· | ·· |
| Weight (kg), mean (SD) | 4.82 (0.639) | 4.72 (0.586) | 4.80 (0.570) | 4.82 (0.598) | 0.7921^b^ | ·· | ·· | ·· |
| Per protocol set |  |  |  |  |  |  |  |  |
| Participants, no. | 83 | 83 | 83 | 83 | ·· | ·· | ·· | ·· |
| Age (days), mean (SD) | 48.5 (4.19) | 48.0 (4.39) | 49.0 (4.17) | 48.1 (4.35) | 0.3446^b^ | ·· | ·· | ·· |
| Sex |  |  |  |  | 0.4852^c^ |  |  |  |
| Male, no. (%) | 44 (53.0%) | 36 (43.4%) | 40 (48.2%) | 45 (54.2%) | ·· | ·· | ·· | ·· |
| Female, no. (%) | 39 (47.0%) | 47 (56.6%) | 43 (51.8%) | 38 (45.8%) | ·· | ·· | ·· | ·· |
| Height (cm), mean (SD) | 55.25 (2.197) | 55.37 (2.049) | 55.65 (1.955) | 55.71 (2.447) | 0.3483^b^ | ·· | ·· | ·· |
| Weight (kg), mean (SD) | 4.83 (0.636) | 4.71 (0.585) | 4.80 (0.571) | 4.82 (0.598) | 0.6888^b^ | ·· | ·· | ·· |
| Sabin type 1 |  |  |  |  |  |  |  |  |
| Seropositive rate, no. (%) | 45 (54.2%) | 46 (55.4%) | 40 (48.2%) | 42 (50.6%) | ·· | 3.6 (−11.3 to 18.3) | 4.8 (−10.2 to 19.5) | 2.4 (−12.5 to 17.2) |
| GMT | 18.62 | 17.69 | 13.18 | 15.69 | ·· | 1.19^a^ (0.79 to 1.79) | 1.13^a^ (0.75 to 1.70) | 0.84^a^ (0.56 to 1.27) |
| Mean Log_2_ titers (SD) | 4.22 (2.224) | 4.14 (2.092) | 3.72 (1.655) | 3.97 (1.755) | ·· | 0.25 (−0.35 to 0.84) | 0.17 (−0.42 to 0.77) | −0.25 (−0.85 to 0.34) |
| Sabin type 2 |  |  |  |  |  |  |  |  |
| Seropositive rate, no. (%) | 45 (54.2%) | 36 (43.4%) | 36 (43.4%) | 38 (45.8%) | ·· | 8.4 (−6.7 to 23.0) | −2.4 (−17.1 to 12.5) | 2.4 (−12.5 to 17.1) |
| GMT | 11.05 | 10.27 | 10.41 | 10.12 | ·· | 1.09^a^ (0.84 to 1.42) | 1.02^a^ (0.78 to 1.32) | 1.03^a^ (0.79 to 1.34) |
| Mean Log_2_ titers (SD) | 3.47 (1.093) | 3.36 (1.373) | 3.38 (1.279) | 3.34 (1.224) | ·· | 0.13 (−0.25 to 0.51) | 0.02 (−0.36 to 0.40) | 0.04 (−0.34 to 0.42) |
| Sabin type 3 |  |  |  |  |  |  |  |  |
| Seropositive rate, no. (%) | 22 (26.5%) | 17 (20.5%) | 26 (31.3%) | 20 (24.1%) | ·· | 2.4 (−10.8 to 15.5) | −3.6 (−16.2 to 9.1) | 7.2 (−6.4 to 20.5) |
| GMT | 9.06 | 8.26 | 8.90 | 8.81 | ·· | 1.03^a^ (0.77 to 1.38) | 0.94^a^ (0.70 to 1.26) | 1.01^a^ (0.75 to 1.36) |
| Mean Log_2_ titers (SD) | 3.18 (1.506) | 3.05 (1.170) | 3.15 (1.307) | 3.14 (1.533) | ·· | 0.04 (−0.38 to 0.46) | −0.09 (−0.52 to 0.33) | 0.02 (−0.41 to 0.44) |
| Wild type 1 (Mahoney) |  |  |  |  |  |  |  |  |
| Seropositive rate, no. (%) | 27 (32.5%) | 20 (24.1%) | 19 (22.9%) | 17 (20.5%) | ·· | 12.0 (−1.4 to 25.0) | 3.6 (−9.1 to 16.2) | −2.4 (−14.9 to 10.1) |
| GMT | 9.41 | 8.37 | 7.86 | 7.27 | ·· | 1.30^a^ (1.01 to 1.66) | 1.15^a^ (0.90 to 1.47) | 1.08^a^ (0.85 to 1.38) |
| Mean Log_2_ titers (SD) | 3.23 (1.438) | 3.07 (1.174) | 2.97 (1.123) | 2.86 (0.843) | ·· | 0.37 (0.02 to 0.73) | 0.20 (−0.15 to 0.56) | 0.11 (−0.24 to 0.47) |
| Wild type 2 (MEF-1) |  |  |  |  |  |  |  |  |
| Seropositive rate, no. (%) | 44 (53.0%) | 36 (43.4%) | 48 (57.8%) | 43 (51.8%) | ·· | 1.2 (−13.7 to 16.0) | −8.4 (−23.0 to 6.6) | −6.0 (−20.6 to 8.9) |
| GMT | 13.18 | 11.73 | 15.78 | 11.86 | ·· | 1.11^a^ (0.80 to 1.55) | 0.99^a^ (0.71 to 1.38) | 1.33^a^ (0.96 to 1.85) |
| Mean Log_2_ titers (SD) | 3.72 (1.505) | 3.55 (1.598) | 3.98 (1.624) | 3.57 (1.504) | ·· | 0.15 (−0.32 to 0.63) | −0.02 (−0.49 to 0.46) | 0.41 (−0.06 to 0.89) |
| Wild type 3 (Saukett) |  |  |  |  |  |  |  |  |
| Seropositive rate, no. (%) | 13 (15.7%) | 10 (12.0%) | 10 (12.0%) | 14 (16.9%) | ·· | −1.2 (−12.6 to 10.2) | −4.8 (−15.7 to 6.1) | −4.8 (−15.7 to 6.1) |
| GMT | 7.66 | 6.80 | 6.57 | 7.51 | ·· | 1.02^a^ (0.82 to 1.27) | 0.90^a^ (0.73 to 1.12) | 0.87^a^ (0.70 to 1.09) |
| Mean Log_2_ titers (SD) | 2.94 (1.277) | 2.76 (0.848) | 2.72 (0.636) | 2.91 (1.191) | ·· | 0.03 (−0.28 to 0.34) | −0.14 (−0.46 to 0.17) | −0.19 (−0.50 to 0.12) |

Abbreviations: sIPV, inactivated polio vaccine made from Sabin strains; cIPV, conventional inactivated polio vaccine; GMT, geometric mean titer; CI, confidence interval; SD, standard deviation.

^a^ The ratio of GMT was calculated as the GMT in the sIPV group divided by the GMT in the cIPV group.

^b^ *P*-value among the four study groups was obtained from Kruskal Wallis test.

^c^ *P*-value among the four study groups was obtained from Chi-square test.
