# Supplementary material for: Safety and Immunogenicity of a New Inactivated Polio Vaccine Made From Sabin Strains: A Randomized, Double-Blind, Active-Controlled, Phase 2/3 Seamless Study
Source: J Infect Dis. 2020 Dec 22;226(2):308–18. doi: 10.1093/infdis/jiaa770 (PMC9400411; doi:10.1093/infdis/jiaa770)
Supplement: jiaa770_suppl_Supplementary_Table_S2 [file jiaa770_suppl_supplementary_table_s2.docx]

## **Table S2. Immunogenicity Results in Stage I (Per Protocol Set)**

|  | **sIPV** | | | **cIPV**  **(N=83)** | **Treatment difference or ratio of GMT^a^ (95% CI)** | | |
| --- | --- | --- | --- | --- | --- | --- | --- |
|  | **Low-dose sIPV**  **(N=83)** | **Middle-dose sIPV**  **(N=83)** | **High-dose sIPV**  **(N=83)** |  | **Low-dose sIPV vs cIPV** | **Middle-dose sIPV vs cIPV** | **High-dose sIPV vs cIPV** |
| Sabin type 1 |  |  |  |  |  |  |  |
| After the second vaccination |  |  |  |  |  |  |  |
| Seroconversion rate, n (%) | 70 (85.4%) | 71 (85.5%) | 77 (92.8%) | 80 (96.4%) | −11.0 (−20.5 to −2.1) | −10.8 (−20.3 to −2.0) | −3.6 (−11.6 to 3.9) |
| GMT | 582.94 | 562.81 | 778.45 | 479.55 | 1.22^a^ (0.81 to 1.83) | 1.17^a^ (0.78 to 1.77) | 1.62^a^ (1.08 to 2.45) |
| GMT ratio^b^ (95% CI) | 32.83 (20.10 to 53.62) | 31.82 (19.54 to 51.81) | 59.04 (36.26 to 96.14) | 30.57 (18.77 to 49.77) | ·· | ·· | ·· |
| Mean Log_2_ titers (SD) | 9.19 (2.166) | 9.14 (1.991) | 9.60 (1.927) | 8.91 (1.627) | 0.28 (−0.31 to 0.87) | 0.23 (−0.36 to 0.82) | 0.70 (0.11 to 1.29) |
| After the third vaccination |  |  |  |  |  |  |  |
| Seroconversion rate, n (%) | 81 (97.6%) | 82 (98.8%) | 83 (100%) | 81 (97.6%) | 0 (−6.2 to 6.2) | 1.2 (−4.4 to 7.2) | 2.4 (−2.3 to 8.4) |
| GMT | 1099.70 | 1188.71 | 1336.03 | 637.17 | 1.73^a^ (1.43 to 2.09) | 1.87^a^ (1.54 to 2.25) | 2.10^a^ (1.74 to 2.53) |
| GMT ratio^b^ (95% CI) | 59.06 (41.94 to 83.17) | 67.20 (47.72 to 94.64) | 101.34 (71.96 to 142.70) | 40.61 (28.84 to 57.19) | ·· | ·· | ·· |
| Mean Log_2_ titers (SD) | 10.10 (0.882) | 10.22 (0.738) | 10.38 (0.449) | 9.32 (1.295) | 0.79 (0.51 to 1.06) | 0.90 (0.63 to 1.17) | 1.07 (0.80 to 1.34) |
| Sabin type 2 |  |  |  |  |  |  |  |
| After the second vaccination |  |  |  |  |  |  |  |
| Seroconversion rate, n (%) | 75 (91.5%) | 79 (95.2%) | 81 (97.6%) | 77 (92.8%) | −1.3 (−10.2 to 7.5) | 2.4 (−5.5 to 10.6) | 4.8 (−2.3 to 12.7) |
| GMT | 262.62 | 482.93 | 587.05 | 163.39 | 1.61^a^ (1.04 to 2.48) | 2.96^a^ (1.92 to 4.55) | 3.59^a^ (2.34 to 5.53) |
| GMT ratio^b^ (95% CI) | 23.78 (15.63 to 36.17) | 47.01 (30.98 to 71.32) | 56.40 (37.17 to 85.58) | 16.15 (10.64 to 24.50) | ·· | ·· | ·· |
| Mean Log_2_ titers (SD) | 8.04 (2.265) | 8.92 (1.969) | 9.20 (1.791) | 7.35 (2.081) | 0.68 (0.06 to 1.31) | 1.56 (0.94 to 2.18) | 1.85 (1.22 to 2.47) |
| After the third vaccination |  |  |  |  |  |  |  |
| Seroconversion rate, n (%) | 83 (100%) | 82 (98.8%) | 83 (100%) | 83 (100%) | 0 (··) | −1.2 (−6.5 to 3.3) | 0 (··) |
| GMT | 660.69 | 1048.66 | 1225.81 | 308.92 | 2.14^a^ (1.70 to 2.69) | 3.39^a^ (2.70 to 4.28) | 3.97^a^ (3.15 to 5.00) |
| GMT ratio^b^ (95% CI) | 59.80 (45.68 to 78.28) | 102.08 (77.97 to 133.63) | 117.77 (89.96 to 154.18) | 30.53 (23.32 to 39.96) | ·· | ·· | ·· |
| Mean Log_2_ titers (SD) | 9.37 (1.180) | 10.03 (0.841) | 10.26 (0.492) | 8.27 (1.552) | 1.10 (0.76 to 1.43) | 1.76 (1.43 to 2.10) | 1.99 (1.66 to 2.32) |
| Sabin type 3 |  |  |  |  |  |  |  |
| After the second vaccination |  |  |  |  |  |  |  |
| Seroconversion rate, n (%) | 79 (96.3%) | 82 (98.8%) | 83 (100%) | 79 (95.2%) | 1.2 (−6.0 to 8.5) | 3.6 (−2.4 to 10.6) | 4.8 (−0.5 to 11.7) |
| GMT | 1044.19 | 1185.24 | 1313.90 | 626.41 | 1.67^a^ (1.27 to 2.19) | 1.89^a^ (1.44 to 2.49) | 2.10^a^ (1.60 to 2.75) |
| GMT ratio^b^ (95% CI) | 122.67 (89.75 to 167.67) | 143.52 (105.20 to 195.79) | 147.56 (108.16 to 201.31) | 71.14 (52.14 to 97.05) | ·· | ·· | ·· |
| Mean Log_2_ titers (SD) | 10.03 (1.263) | 10.21 (1.063) | 10.36 (0.570) | 9.29 (1.893) | 0.74 (0.34 to 1.13) | 0.92 (0.53 to 1.31) | 1.07 (0.68 to 1.46) |
| After the third vaccination |  |  |  |  |  |  |  |
| Seroconversion rate, n (%) | 82 (98.8%) | 83 (100%) | 83 (100%) | 81 (97.6%) | 1.2 (−4.4 to 7.2) | 2.4 (−2.3 to 8.4) | 2.4 (−2.3 to 8.4) |
| GMT | 1310.28 | 1412.56 | 1432.28 | 740.40 | 1.77^a^ (1.54 to 2.03) | 1.91^a^ (1.66 to 2.19) | 1.93^a^ (1.69 to 2.22) |
| GMT ratio^b^ (95% CI) | 144.69 (112.12 to 186.73) | 171.04 (132.54 to 220.73) | 160.86 (124.65 to 207.59) | 84.08 (65.15 to 108.51) | ·· | ·· | ·· |
| Mean Log_2_ titers (SD) | 10.36 (0.495) | 10.46 (0.164) | 10.48 (0.071) | 9.53 (1.176) | 0.82 (0.63 to 1.02) | 0.93 (0.74 to 1.13) | 0.95 (0.76 to 1.15) |
| Wild type 1 (Mahoney) |  |  |  |  |  |  |  |
| After the second vaccination |  |  |  |  |  |  |  |
| Seroconversion rate, n (%) | 60 (73.2%) | 58 (69.9%) | 69 (83.1%) | 81 (97.6%) | −24.4 (−35.0 to −14.1) | −27.7 (−38.4 to −17.1) | −14.5 (−24.1 to −5.6) |
| GMT | 38.37 | 35.86 | 49.40 | 653.39 | 0.06^a^ (0.04 to 0.09) | 0.05^a^ (0.03 to 0.09) | 0.08^a^ (0.05 to 0.12) |
| GMT ratio^b^ (95% CI) | 4.11 (2.74 to 6.16) | 4.28 (2.86 to 6.40) | 6.29 (4.21 to 9.40) | 89.91 (60.14 to 134.40) | ·· | ·· | ·· |
| Mean Log_2_ titers (SD) | 5.26 (2.429) | 5.16 (2.286) | 5.63 (2.255) | 9.35 (1.507) | −4.09 (−4.75 to −3.43) | −4.19 (−4.84 to −3.53) | −3.73 (−4.38 to −3.07) |
| After the third vaccination |  |  |  |  |  |  |  |
| Seroconversion rate, n (%) | 75 (90.4%) | 77 (92.8%) | 81 (97.6%) | 83 (100%) | −9.6 (−17.9 to −3.2) | −7.2 (−14.9 to −1.3) | −2.4 (−8.4 to 2.3) |
| GMT | 90.90 | 122.80 | 191.39 | 703.34 | 0.13^a^ (0.08 to 0.20) | 0.17^a^ (0.11 to 0.27) | 0.27^a^ (0.18 to 0.42) |
| GMT ratio^b^ (95% CI) | 9.66 (6.71 to 13.89) | 14.66 (10.19 to 21.10) | 24.36 (16.93 to 35.05) | 96.78 (67.26 to 139.25) | ·· | ·· | ·· |
| Mean Log_2_ titers (SD) | 6.51 (2.143) | 6.94 (2.406) | 7.58 (2.036) | 9.46 (1.184) | −2.95 (−3.56 to −2.34) | −2.52 (−3.13 to −1.91) | −1.88 (−2.49 to −1.27) |
| Wild type 2 (MEF-1) |  |  |  |  |  |  |  |
| After the second vaccination |  |  |  |  |  |  |  |
| Seroconversion rate, n (%) | 67 (81.7%) | 72 (86.7%) | 77 (92.8%) | 78 (94.0%) | −12.3 (−22.6 to −2.2) | −7.2 (−16.8 to 2.0) | −1.2 (−9.6 to 7.1) |
| GMT | 197.54 | 362.04 | 527.23 | 564.32 | 0.35^a^ (0.22 to 0.57) | 0.64^a^ (0.40 to 1.04) | 0.93^a^ (0.58 to 1.51) |
| GMT ratio^b^ (95% CI) | 14.83 (8.95 to 24.59) | 30.87 (18.68 to 51.02) | 33.42 (20.22 to 55.23) | 47.58 (28.79 to 78.63) | ·· | ·· | ·· |
| Mean Log_2_ titers (SD) | 7.63 (2.530) | 8.50 (2.445) | 9.04 (2.126) | 9.14 (1.957) | −1.51 (−2.21 to −0.82) | −0.64 (−1.34 to 0.05) | −0.10 (−0.79 to 0.60) |
| After the third vaccination |  |  |  |  |  |  |  |
| Seroconversion rate, n (%) | 79 (95.2%) | 81 (97.6%) | 83 (100%) | 82 (98.8%) | −3.6 (−10.6 to 2.4) | −1.2 (−7.2 to 4.4) | 1.2 (−3.3 to 6.5) |
| GMT | 407.55 | 643.43 | 961.99 | 899.67 | 0.45^a^ (0.34 to 0.60) | 0.72^a^ (0.54 to 0.94) | 1.07^a^ (0.81 to 1.41) |
| GMT ratio^b^ (95% CI) | 30.92 (21.86 to 43.73) | 54.86 (38.79 to 77.60) | 60.97 (43.11 to 86.24) | 75.85 (53.62 to 107.28) | ·· | ·· | ·· |
| Mean Log_2_ titers (SD) | 8.67 (1.772) | 9.33 (1.380) | 9.91 (0.877) | 9.81 (0.941) | −1.14 (−1.54 to −0.75) | −0.48 (−0.88 to −0.09) | 0.10 (−0.30 to 0.49) |
| Wild type 3 (Saukett) |  |  |  |  |  |  |  |
| After the second vaccination |  |  |  |  |  |  |  |
| Seroconversion rate, n (%) | 78 (95.1%) | 82 (98.8%) | 83 (100%) | 79 (95.2%) | −0.1 (−7.7 to 7.5) | 3.6 (−2.4 to 10.6) | 4.8 (−0.5 to 11.7) |
| GMT | 680.69 | 990.36 | 1102.55 | 498.71 | 1.36^a^ (0.95 to 1.96) | 1.99^a^ (1.38 to 2.85) | 2.21^a^ (1.54 to 3.17) |
| GMT ratio^b^ (95% CI) | 94.70 (68.11 to 131.65) | 145.70 (105.01 to 202.16) | 167.72 (120.88 to 232.71) | 66.36 (47.83 to 92.08) | ·· | ·· | ·· |
| Mean Log_2_ titers (SD) | 9.41 (1.990) | 9.95 (1.468) | 10.11 (1.023) | 8.96 (2.110) | 0.45 (−0.07 to 0.97) | 0.99 (0.47 to 1.51) | 1.14 (0.62 to 1.66) |
| After the third vaccination |  |  |  |  |  |  |  |
| Seroconversion rate, n (%) | 82 (98.8%) | 83 (100%) | 83 (100%) | 82 (98.8%) | 0 (−5.4 to 5.4) | 1.2 (−3.3 to 6.5) | 1.2 (−3.3 to 6.5) |
| GMT | 961.99 | 1251.57 | 1321.27 | 628.19 | 1.53^a^ (1.24 to 1.90) | 1.99^a^ (1.61 to 2.47) | 2.10^a^ (1.70 to 2.61) |
| GMT ratio^b^ (95% CI) | 125.54 (98.51 to 159.99) | 184.13 (144.48 to 234.65) | 200.99 (157.71 to 256.14) | 83.59 (65.59 to 106.53) | ·· | ·· | ·· |
| Mean Log_2_ titers (SD) | 9.91 (1.182) | 10.29 (0.480) | 10.37 (0.312) | 9.30 (1.541) | 0.61 (0.31 to 0.92) | 0.99 (0.69 to 1.30) | 1.07 (0.76 to 1.38) |

Abbreviations: sIPV, inactivated polio vaccine made from Sabin strains; cIPV, conventional inactivated polio vaccine; GMT, geometric mean titer; CI, confidence interval; SD, standard deviation.

^a^ The ratio of GMT was calculated as the GMT in the sIPV group divided by the GMT in the cIPV group.

^b^ GMT ratio was calculated as the GMT at the post-vaccination divided by the GMT at the pre-vaccination.
