# Supplementary material for: Safety and Immunogenicity of a New Inactivated Polio Vaccine Made From Sabin Strains: A Randomized, Double-Blind, Active-Controlled, Phase 2/3 Seamless Study
Source: J Infect Dis. 2020 Dec 22;226(2):308–18. doi: 10.1093/infdis/jiaa770 (PMC9400411; doi:10.1093/infdis/jiaa770)
Supplement: jiaa770_suppl_Supplementary_Table_S3 [file jiaa770_suppl_supplementary_table_s3.docx]

## **Table S3. Adverse Events in Stage I (Safety Set)**

|  | **sIPV** | | | | **cIPV (N=83) no. (%)** | **Overall (N=335) no. (%)** |
| --- | --- | --- | --- | --- | --- | --- |
|  | **Low-dose sIPV (N=84) no. (%)** | **Middle-dose sIPV (N=84) no. (%)** | **High-dose sIPV (N=84) no. (%)** | **Combined sIPV (N=252) no. (%)** |  |  |
| Any adverse events up to close-out visit^a^ | 70 (83.3%) | 66 (78.6%) | 75 (89.3%) | 211 (83.7%) | 66 (79.5%) | 277 (82.7%) |
| % Difference vs cIPV (95% CI) | 3.8 (−8.1 to 15.6) | −0.9 (−13.3 to 11.4) | 9.8 (−1.4 to 20.8) | 4.2 (−4.7 to 14.9) | ·· | ·· |
| Immediate reactions | 0 | 0 | 0 | 0 | 0 | 0 |
| Solicited adverse events | 63 (75.0%) | 61 (72.6%) | 72 (85.7%) | 196 (77.8%) | 52 (62.7%) | 248 (74.0%) |
| % Difference vs cIPV (95% CI) | 12.3 (−1.7 to 25.8) | 10 (−4.2 to 23.6) | 23.1 (9.9 to 35.3) | 15.1 (4.0 to 26.9) | ·· | ·· |
| Solicited adverse drug reactions | 62 (73.8%) | 61 (72.6%) | 72 (85.7%) | 195 (77.4%) | 49 (59.0%) | 244 (72.8%) |
| % Difference vs cIPV (95% CI) | 14.8 (0.5 to 28.3) | 13.6 (−0.8 to 27.2) | 26.7 (13.2 to 39.0) | 18.3 (6.9 to 30.1) | ·· | ·· |
| Solicited local adverse events | 42 (50.0%) | 37 (44.0%) | 41 (48.8%) | 120 (47.6%) | 28 (33.7%) | 148 (44.2%) |
| Pain/Tenderness | 39 (46.4%) | 33 (39.3%) | 40 (47.6%) | 112 (44.4%) | 27 (32.5%) | 139 (41.5%) |
| Erythema/Redness | 7 (8.3%) | 7 (8.3%) | 5 (6.0%) | 19 (7.5%) | 3 (3.6%) | 22 (6.6%) |
| Induration/Swelling | 4 (4.8%) | 2 (2.4%) | 3 (3.6%) | 9 (3.6%) | 1 (1.2%) | 10 (3.0%) |
| Solicited systemic adverse events | 57 (67.9%) | 57 (67.9%) | 67 (79.8%) | 181 (71.8%) | 49 (59.0%) | 230 (68.7%) |
| Fever | 6 (7.1%) | 12 (14.3%) | 22 (26.2%) | 40 (15.9%) | 3 (3.6%) | 43 (12.8%) |
| Irritability/Restlessness | 45 (53.6%) | 45 (53.6%) | 52 (61.9%) | 142 (56.3%) | 37 (44.6%) | 179 (53.4%) |
| Drowsiness/Sleepiness | 29 (34.5%) | 32 (38.1%) | 36 (42.9%) | 97 (38.5%) | 26 (31.3%) | 123 (36.7%) |
| Loss of appetite | 9 (10.7%) | 12 (14.3%) | 17 (20.2%) | 38 (15.1%) | 11 (13.3%) | 49 (14.6%) |
| Diarrhea | 19 (22.6%) | 16 (19.0%) | 13 (15.5%) | 48 (19.0%) | 12 (14.5%) | 60 (17.9%) |
| Vomiting | 8 (9.5%) | 12 (14.3%) | 14 (16.7%) | 34 (13.5%) | 10 (12.0%) | 44 (13.1%) |
| Rash | 5 (6.0%) | 8 (9.5%) | 6 (7.1%) | 19 (7.5%) | 4 (4.8%) | 23 (6.9%) |
| Unsolicited adverse events | 39 (46.4%) | 39 (46.4%) | 30 (35.7%) | 108 (42.9%) | 47 (56.6%) | 155 (46.3%) |
| % Difference vs cIPV (95% CI) | −10.2 (−24.6 to 4.9) | −10.2 (−24.6 to 4.9) | −20.9 (−34.7 to −5.8) | −13.8 (−25.5 to −1.4) | ·· | ·· |
| Unsolicited adverse drug reactions | 0 | 0 | 1 (1.2%) | 1 (0.4%) | 0 | 1 (0.3%) |
| % Difference vs cIPV (95% CI) | 0 (··) | 0 (··) | 1.2 (−3.3 to 6.4) | 0.4 (−4.0 to 2.2) | ·· | ·· |
| Any serious adverse events up to close-out visit^a^ | 1 (1.2%) | 4 (4.8%) | 2 (2.4%) | 7 (2.8%) | 3 (3.6%) | 10 (3.0%) |
| % Difference vs cIPV (95% CI) | −2.4 (−9.0 to 3.3) | 1.1 (−6.0 to 8.4) | −1.2 (−7.9 to 5.1) | −0.8 (−7.5 to 2.9) | ·· | ·· |
| Solicited serious adverse events | 0 | 0 | 0 | 0 | 0 | 0 |
| Unsolicited serious adverse events | 1 (1.2%) | 4 (4.8%) | 2 (2.4%) | 7 (2.8%) | 3 (3.6%) | 10 (3.0%) |
| Unsolicited serious adverse drug reactions | 0 | 0 | 0 | 0 | 0 | 0 |
| Any serious adverse events after close-out visit^a^ | 2 (2.4%) | 5 (6.0%) | 3 (3.6%) | 10 (4.0%) | 1 (1.2%) | 11 (3.3%) |
| % Difference vs cIPV (95% CI) | 1.2 (−4.4 to 7.1) | 4.7 (−1.5 to 12.1) | 2.4 (−3.4 to 8.9) | 2.8 (−2.8 to 6.1) | ·· | ·· |

Abbreviations: sIPV, inactivated polio vaccine made from Sabin strains; cIPV, conventional inactivated polio vaccine; CI, confidence interval.

^a^ The close-out visit was 1 month after the last vaccination.
